# Development of a Guidance System for Motor Imagery Enhancement Using the Virtual Hand Illusion

**DOI:** 10.3390/s21062197

**Published:** 2021-03-21

**Authors:** Hojun Jeong, Jonghyun Kim

**Affiliations:** School of Mechanical Engineering, Sungkyunkwan University, Suwon 16419, Korea; ghwns1664@skku.edu

**Keywords:** motor imagery, brain-computer interface, guidance system, virtual hand illusion, virtual reality, motion tracking sensor, event-related desynchronization

## Abstract

Motor imagery (MI) is widely used to produce input signals for brain–computer interfaces (BCI) due to the similarities between MI-BCI and the planning–execution cycle. Despite its usefulness, MI tasks can be ambiguous to users and MI produces weaker cortical signals than motor execution. Existing MI guidance systems, which have been reported to provide visual guidance for MI and enhance MI, still have limitations: insufficient immersion for MI or poor expandability to MI for another body parts. We propose a guidance system for MI enhancement that can immerse users in MI and will be easy to extend to other body parts and target motions with few physical constraints. To make easily extendable MI guidance system, the virtual hand illusion is applied to the MI guidance system with a motion tracking sensor. MI enhancement was evaluated in 11 healthy people by comparison with another guidance system and conventional motor commands for BCI. The results showed that the proposed MI guidance system produced an amplified cortical signal compared to pure MI (*p* < 0.017), and a similar cortical signal as those produced by both actual execution (*p* > 0.534) and an MI guidance system with the rubber hand illusion (*p* > 0.722) in the contralateral region. Therefore, we believe that the proposed MI guidance system with the virtual hand illusion is a viable alternative to existing MI guidance systems in various applications with MI-BCI.

## 1. Introduction

Motor imagery (MI) is defined as the mental rehearsal of a movement without any actual movement or muscle activation [1]. It results in a decrease in power of sensory-motor rhythms, called event-related desynchronization (ERD), where the decrease in brain potential usually occurs before the onset of motion [2,3]. Based on this phenomenon, MI ERD has been used to predict motion or movement intention in the brain–computer interface (BCI) field [4,5]. By predicting the user’s intention, BCI technologies can provide the user with appropriate feedback at the intended time. Likewise, due to the similarity of the planning–execution cycle, MI-BCI is widely used for rehabilitation and real-life applications [6,7]. Although MI ERD shares the frequency band and area with ERD from motor execution (ME) [8,9], it produces significantly less brain cortical activity than ME ERD [10]. The weak electrical signal from this low-level cortical activation leads to difficulties in MI-BCI classification and causes poor performance of the classification results [11,12].

To solve this problem, several attempts have been made to enhance MI with guidance systems [13,14,15,16]. Action observation (AO), which elicits similar activity in the motor system as MI, is used to enhance MI by displaying target motions and guiding users visually. The combination of AO and MI results in significantly higher activation than pure AO/MI [13,14,15]. In addition, this system has the advantage of being simple and easy to apply, with few physical constraints. In a few previous studies that applied virtual reality (VR) to immersive MI, VR-based AO resulted in amplification of ERD and enhanced intention detection [15,16]. Moreover, there were several attempts to induce ownership to virtual body parts and embody MI by applying VR-based AO to the feedback of MI-BCI [17,18,19], and some of them provided additional information for more immersive MI [19].

Another attempt to enhance MI involves application of the rubber hand illusion (RHI) for the MI guidance system [20]. The RHI induces a feeling of ownership of the fake body part, such that the fake body part feels like the user’s own body part [21]. The philosophy of the RHI-based MI guidance system is an attempt to induce body ownership and increase the sense of immersion through multisensory integration (vision, proprioceptive, etc.) beyond the previous AO-based MI guidance system. The paradigm for enhancing MI with RHI consists of two main parts: first, providing the illusion to induce ownership of the rubber hand; and second, providing visual guidance for the target movement [20]. The main difference between AO-based and RHI-based MI guidance systems is that, by inducing body ownership, the target motion provided by the moving hand creates the illusion of moving the user’s own hand. Our previous study reported that the RHI-based MI guidance system shows significantly higher ERD amplitude than AO-based guidance as well as pure MI [20].

However, unlike MI guidance with AO, RHI requires a physical system to induce ownership of specific body parts. This requirement limits the ability to extend the illusion to other body parts and target motions, and it makes this system difficult to apply in various contexts [22]. Furthermore, due to the physical presence of the fake hands, there is an inevitable gap between the (fake) rubber body parts and the user’s own body parts. This results in a discrepancy between visual and proprioceptive information, which can decrease the user’s sense of ownership of the fake hand [23].

To overcome this constraint of RHI, the virtual hand illusion (VHI) has been used as a body ownership illusion method in recent years [24,25,26]. VHI is easier to extend to other body parts or complex target motions than RHI thanks to its lack of physical constraints. Moreover, VHI can reduce the perceived gap between proprioception and position. Additionally, using a motion-tracking sensor, the position of the user’s own hand is tracked; the system displays the position of the virtual hand based on the information from the sensor [26].

Based on the VHI system, the present study proposes an MI guidance system with VHI. Our proposed system is verified by quantitative evaluation of the resulting MI enhancement. To that end, (1) We implemented an easily extendable MI guidance system using VHI, which consists of two main components, generating the illusion and providing guidance, for immersive MI; and (2) We compared the MI guidance system with VHI to the system with RHI and two conventional motor commands for BCI, MI, and ME. The evaluation was performed in eleven healthy subjects. The proposed guidance system using VHI shows higher activation than MI and results that are comparable to those of RHI.

## 2. Methods

### 2.1. Proposed Guidance System for MI Enhancement

The illusion-based MI guidance is divided into two sessions: (1) providing a proper environment with stimuli such that body ownership can be induced (illusion session), and (2) providing the user with a target motion to imagine (imaginary session). For the guidance, we implemented RHI-based and VHI-based MI guidance systems.

**Figure 1:**
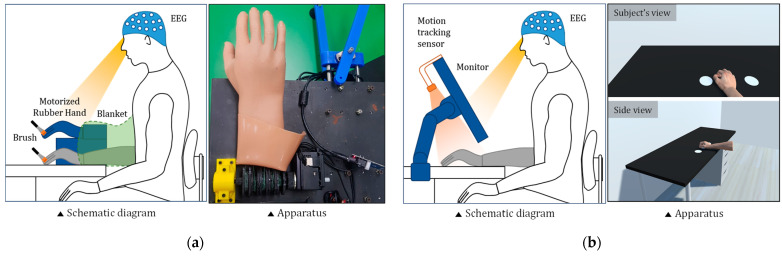
Schematic diagram and apparatus for (**a**) rubber hand illusion (RHI) and (**b**) virtual hand illusion (VHI). The gray hand denotes the subject’s real hand, and the navy blue objects are the systems which the subject observes for MI enhancement.

For the RHI-based MI guidance system, the box was positioned on a table and a life-sized model hand was placed on top of the box. The subjects placed their right hand inside the box; their hand and forearm were blocked from view, as were the actuating parts of the moving rubber hand, with a cloth (Figure 1a). All subjects were instructed to observe the rubber hand during all sessions. In the illusion session, the operator used brushes to stroke both the rubber hand and the user’s own hand in the same location simultaneously. During a session, the subject feels as though the rubber hand is their own hand (feeling of ownership) because the brush stroking stimuli is felt on their own hand and visually observed on the rubber hand. After the illusion session, the motorized rubber hand was activated for the imagination session, as illustrated in Figure 1a. In the imagination session, the subjects were asked to perform MI by observing the movement of the rubber hand and could perform MI more easily by watching the rubber hand perform the target motion. Note that the RHI-based MI guidance system is a modified version of [20].

For the VHI-based MI guidance system, a life-sized virtual hand in virtual space was implemented by the graphical engine. The subject positioned their hand behind the monitor on the desk and watched the virtual space on the monitor in front of them instead of viewing the actual hand, as illustrated in Figure 1b. Motion tracking sensors tracked the user’s hand position to match the virtual hand position with that of the user’s blocked hand. The subjects were instructed to observe the virtual hand during all sessions, as in the protocols of the RHI-based MI guidance system. In the illusion session, we used the virtual hand illusion method modified in [26]. The subjects performed reaching tasks to randomly presented virtual targets using a virtual hand model with an attached forearm. After the illusion session, the virtual hand performed target motion during the imagination session. In the imagination session, all procedures were the same as in the imagination session for the RHI-based MI guidance system, except the fake hand was not a rubber hand but a virtual hand.

### 2.2. Experimental Designs

Experimental comparison was conducted to evaluate the degree of MI enhancement for the proposed VHI-based MI guidance system. The proposed VHI-based MI guidance system was compared with another guidance system, the RHI-based MI guidance system, and two conventional motor commands for BCI, pure MI, and pure ME. A paradigm for pure MI (MI-P) was conducted before the other paradigms in order to assess pure MI without the effects of any guidance system. Paradigms for RHI-based MI guidance system (RHI-P) and VHI-based MI guidance system (VHI-P) followed MI-P in random order. A paradigm for pure ME (ME-P) was conducted last so that the ME experience did not affect MI, as it might make the user’s MI significantly easier.

MI-P and ME-P were compared with other guidance systems for MI enhancement. MI-P was designed to evaluate subject’s inherent, pure MI abilities in comparison of MI enhancement from RHI-P and VHI-P, whereas ME-P was performed to compare MI enhancement from RHI-P and VHI-P to actual execution with the assumption that brain cortical signal caused by ME is significantly greater than that caused by MI. To ensure a fair comparison, all paradigms used an auditory cue to inform the start of the task period.

For the experiment, eleven healthy subjects (7 females and 4 males; all right-handed; age: 23.9 ± 3.3 years; six subjects were novice users of BCI) who do not have any history of neurological disorders were recruited for the experiment. This experiment was approved by the Sungkyunkwan University Institutional Review Board (SKKU 2020-08-022), and all subjects voluntarily consented to participate in the experiment in writing after they were informed of the experimental details.

### 2.3. Apparatus

RHI-P used a motorized silicon-based rubber hand with an actuator (MX106R, ROBOTIS, Seoul, Korea), as shown in Figure 1a. To mimic the open/close motion of a normal hand, the force of the actuator was delivered to 3D-printed finger bones using a cable-driven mechanism and the motor was driven based on simple positional control. 

For VHI-P, the components of the VR environment were designed using 3D creative software (Blender, Blender Foundation) and implemented in a graphical engine, Unity 3D (Unity, Unity technologies SF, San Francisco, CA, USA). From a first-person perspective, the virtual environment contained a desk, which was a 1:1 replica of the real desk, with the same shape, size, and position. Moreover, the virtual and real environments were carefully aligned by measuring the location and position. A monitor that displayed the VR environment was positioned in front of the subject using the monitor desk mount, as illustrated in Figure 1b. To track the position of the user’s hand, a motion-tracking sensor (LEAP motion controller, Leap Motion Inc., San Francisco, CA, USA) was attached to the monitor with a custom-made holder.

### 2.4. Experimental Protocols

**Figure 2:**
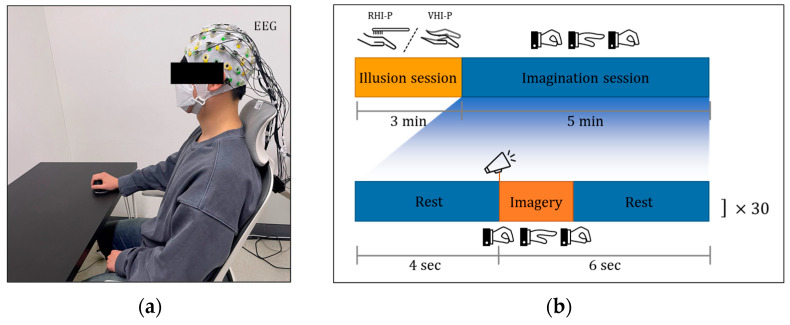
Experimental protocol: (**a**) Setup of pure motor imagery (MI-P) and pure motor execution (ME-P); (**b**) trial design. The upper blocks indicate the protocol for the illusion-based MI guidance system. Each illusion session for VHI-P and RHI-P lasted 3 min, and the imagination session was followed for 5 min. The lower blocks indicate the protocol for MI-P and the imagination session of the illusion-based MI guidance system. In the session, imagery task was performed whenever an auditory cue that was repeated in every 10 s was provided.

The MI-P and ME-P protocols are illustrated in Figure 2. The subject wore an Electroencephalogram (EEG) cap and rested his/her hands comfortably on a custom-designed palm supporter on a table. The subjects were instructed to perform a single hand open/close motion MI or ME at a speed of 0.5 Hz whenever they heard the beeping sound. Here, the motion is that all finger joints extend to the open hand and then return to the rest posture.

As mentioned in Section 2.1, the illusion-based MI guidance systems, RHI-P and VHI-P, consisted of illusion sessions and imagination sessions. To enable a fair comparison, each illusion session (RHI-P and VHI-P) lasted for 3 min, respectively, as illustrated in Figure 2b. After that, the subjects were instructed to observe the single hand open/close motion of the fake hand (i.e., rubber hand in RHI-P and virtual hand in VHI-P) and imagine performing the same motion. Here, the single hand open/close motion of the fake hand was performed at a speed of 0.5 Hz.

For each paradigm, 30 trials were performed, lasting 5 min in total. Subjects were asked to focus on the task and to avoid other conscious mental tasks. About 5 min of rest was provided between paradigms to minimize interference. The entire duration of the experiment was about 45 min (except for the EEG setup). After the experiments, a questionnaire was provided to the subjects so that they could indicate which paradigm induced a greater sense of body ownership (i.e., which fake hand felt more like their own hand) among the illusion-based MI guidance systems. The statements of the questionnaire, a modified version of [21], were as follows:

(1) RHI-P: I felt as if the fake hand in RHI-P were my hand more than that in VHI-P;
(2) VHI-P: I felt as if the fake hand in VHI-P were my hand more than that in RHI-P;
(3) Both: I felt as if the fake hands in both RHI-P and VHI-P were my hand similarly;
(4) None: I could not feel as if either the fake hand in RHI-P or VHI-P were my hand.

### 2.5. Data Acquisition & Analysis

Raw data (500 Hz, 32 channel) was acquired from the EEG device (BrainAmp DC, BrainProduct, GmbH, Gilching, Germany), and EEG electrodes were put on an EEG cap based on the following 10–20 system channel locations: AF3, AF4, F7, F3, Fz, F4, F8, FC5, FC3, FC1, FCz, FC2, FC4, FC6, C5, C3, C1, Cz, C2, C4, C6, CP5, CP3, CP1, CPz, CP2, CP4, CP6, P1, P2, PO3, and PO4, almost of all which focused on motor-related areas.

The raw EEG data was pre-processed as follows. Bad channels were detected based on kurtosis and interpolated by a linear combination of the nearby channels using EEGLAB functions [27]. A band-pass filter (8 to 30 Hz) was applied to extract the mu (8–13 Hz) and beta (14–30Hz) rhythms, which are well-known motor-related frequency bands [8,9,28]. Then, independent component analysis (ICA) was conducted to reject the signals from eye movements and other motion artifacts [29]. We next extracted data epochs (-4 to 6 s from the cue) based on time information in the preprocessed data and subtracted the baseline (-4 to -2 s from the cue). Abnormal epochs (those exceeding ±100 μV), which was about 5% of total epochs, were rejected from further analysis.

For investigating the details on ERD and event-related synchronization (ERS) patterns for different tasks, event-related spectral perturbation (ERSP) was used to represent the average event-related power changes in a time-frequency map. The ERSP of *n* trials was calculated as follow [27]:

(1) $$\mathrm{ERSP}(f,t)=\frac{1}{n}\sum_{k=1}^{n}\left(F_{k}{\left(f,t\right)}^{2}\right)$$

where $F_{k}(f,t)$ represents the spectral estimation value at frequency f and time t for the *k*th trial. EEGLAB functions [27] were used to calculate the ERSP (dB). For the baseline normalized ERSP, the average power changes in the baseline period (-4 to -2 s from the cue) were subtracted from all spectral estimation. In this study, the ERSP of C3, the contralateral channel corresponding to hand motor tasks, was displayed from -4 to 6 s between 4 and 32 Hz, according to the method used to calculate baseline normalized ERSP. To investigate topographic distribution, the average ERSP of the mu and beta bands (8–30 Hz), which showed desynchronized patterns when a motor-related task was performed, was calculated in each channel for the task period.

Furthermore, the relative ERD, percentage of power decrease by the calculation method [30], was extracted to obtain the subject’s relative ERD for each paradigm. Note that the relative ERD was calculated by averaging the epochs performed in each paradigm for each subject [30]. Then, the peak ERD for each paradigm was calculated from the lowest peak of $ERD_{\%}$ to quantitatively investigate MI enhancement. The latency, which represents the temporal characteristics of ERD, was calculated to compare the speed at which the $ERD_{\%}$ reached the negative peak in each paradigm. This latency is an index that can be used to quantify how fast the BCI system could detect ERD [31,32]. For the statistical analysis, the Friedman test (non-parametric test [33]) was performed between the four paradigms for peak ERD and latency, and the Wilcoxon signed-rank test was performed for post-hoc analysis [34]. MI enhancement was evaluated in both the contralateral region (C3 channel) and the ipsilateral region (C4 channel) to assess mirror neuron activation, which is known to be related to performing a motor task or observing motor actions being performed by other people [35,36].

Based on the results of the questionnaires, we investigated the agreement between the intensity of the subject’s feelings for body ownership and the paradigm that results in amplified ERD.

## 3. Results

### 3.1. ERSP Map and Topographical Distrubution

**Figure 3:**
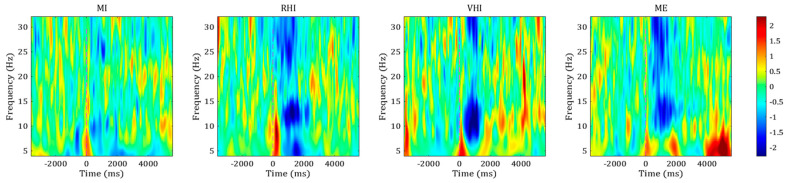
Averaged event-related spectral perturbation (ERSP) maps of each paradigm. The red dotted lines indicate task onset.

Figure 3 shows the averaged time-frequency maps across all participants for the four different paradigms at C3. The ERSP maps represent ERD patterns at task onset for all conditions. The four different paradigms showed desynchronized patterns in the mu and beta bands, in particular, strong ERD patterns from 10 to 15 Hz.

**Figure 4:**
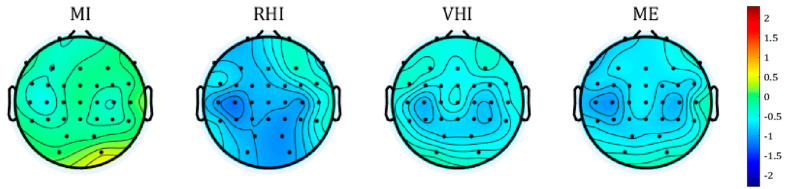
Topographic distribution of average ERSP for each paradigm, within a two-second window after the cue.

According to the topographic distribution, as illustrated in Figure 4, RHI, VHI, and ME showed the strongest ERD signals near the contralateral (C3) and ipsilateral (C4) channel, whereas MI showed weaker activation compared to the other three paradigms.

### 3.2. Analysis of Relative ERD

**Figure 5:**
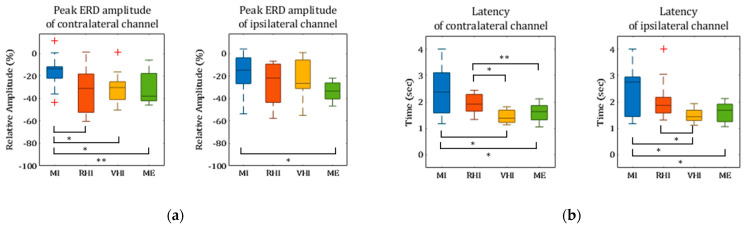
Findings in the contralateral and ipsilateral channel: (**a**) peak event-related desynchronization (ERD) amplitude and (**b**) latency. (The asterisks indicate significant differences, * *p* < 0.05, ** *p* < 0.01).

Figure 5a and Table 1 show the peak ERD amplitude for each paradigm and the statistical differences between paradigms in the C3 channel in the contralateral region and the C4 channel in the ipsilateral region. In the contralateral channel, ME-P showed a significantly larger amplitude than MI-P (*p* < 0.01). RHI-P and VHI-P showed a similar amplitude to ME-P, with a statistically significant difference compared to MI-P (*p* < 0.05). RHI-P and VHI-P did not significantly differ from ME-P (*p* > 0.1). Moreover, VHI-P did not significantly differ from RHI-P (*p* > 0.1). In contrast, in the ipsilateral channel, peak ERD amplitude showed little difference between paradigms (*p* = 0.058). Only ME-P showed a significantly larger amplitude than MI-P.

As shown in Figure 5b and Table 1, in the contralateral channel, latency significantly differed between paradigms (*p* < 0.05). Compared to MI-P and RHI-P, VHI-P and ME-P had significantly smaller latency (*p* < 0.05). However, there are no significant differences between the latencies of MI-P and RHI-P, or between those of VHI-P and ME-P (*p* > 0.1). Like in the contralateral channel, there are significant differences in latency between paradigms in the ipsilateral channel. ME-P showed a significantly smaller latency than MI-P (*p* < 0.05). Furthermore, VHI-P had a significantly smaller latency than MI-P and RHI-P (*p* < 0.05).

### 3.3. Questionnaire

**Figure 6:**
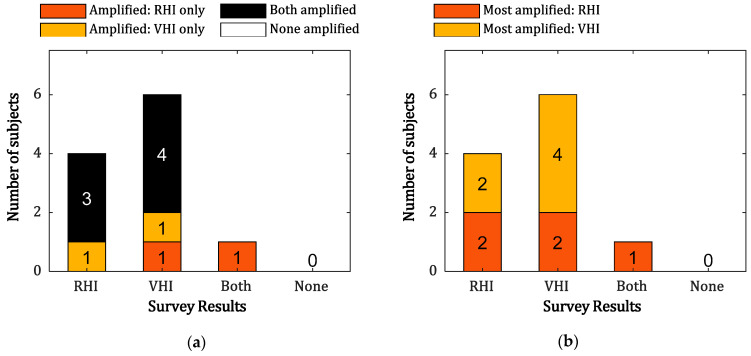
Survey results for each paradigm: (**a**) Amplified MI guidance system vs. MI-P; (**b**) Most amplified MI guidance system. The black block represents the subjects with MI ERD enhancement in both paradigms, while the red and yellow blocks represent the subjects with enhancement in RHI-P and VHI-P, respectively.

Figure 6 summarizes the survey results on the subjects’ intensity of feelings of body ownership in the proposed system compared to the other MI guidance systems. Figure 6a illustrates the relationship between the survey results (i.e., which MI guidance system induced a greater feeling of body ownership) and enhancement of the relative ERD in the MI guidance systems compared to MI-P. On average, seven of 11 subjects showed greater amplification of the ERD in both MI guidance systems than MI-P, whereas four of 11 subjects showed greater amplification of the ERD in only one of the MI guidance systems. All subjects showed MI enhancement in at least one of the MI guidance systems. Among MI guidance systems, VHI-P amplified ERD in six subjects and RHI-P amplified ERD in five subjects (Figure 6b). With the exception of one subject who reported enhancement of body ownership in both systems, 6 out of 10 subjects showed agreement between their survey results and the paradigm that exhibited the greatest amplification of $ERD_{\%}$.

## 4. Discussion

The present study aims to (1) propose guidance systems for MI enhancement that are easy to apply in real-life situations, and (2) evaluate the MI enhancement of the proposed system in comparison to that of other MI guidance systems and motor commands for BCI. To these ends, we proposed an MI guidance system using VHI with a motion-tracking sensor. In our previous work [20], significant MI enhancement was achieved by a RHI-based MI guidance system. In spite of the enhancement, some required setups for the RHI-based system (motorized rubber hand and ownership inducing method by brushes) made the system difficult to apply in various applications with the presence of physical constraints. In contrast, the proposed system in this study has relatively few physical constraints, as it only necessitates a VR device and a motion-tracking sensor, which makes it easy to extend the system to other body parts or target motions. Moreover, the comparative experimental results showed that the proposed system produced significant enhancement of MI ERD despite having so few physical constraints. It should be note that the RHI-based system also has an advantage that it does not require actual movement to induce body ownership illusion. 

The comparison of average ERSP maps in the contralateral channel (Figure 3) showed that the MI guidance systems, as well as MI-P and ME-P, produced a desynchronized pattern in the mu and beta bands, especially in the upper mu band (10–12 Hz) and SMR band (12–15 Hz), for about 2 s after the cue. Since the upper mu band and SMR band are related to motor-related tasks (e.g., movement or observation) [37,38,39], these findings demonstrate that the motor-related tasks were performed well in all of the paradigms. The same trend was observed in the topographic distribution results (Figure 4). All of the paradigms showed strong ERD patterns in bilateral regions, near C3 and C4, where the mu and beta rhythms became desynchronized when the hand-related motor task was performed [39,40,41]. The ERSP maps and topographic distribution results demonstrate that illusion-based MI guidance systems and conventional motor commands were reflected by similar frequency bands (10–15 Hz) and source channels (C3 and C4) which showed the strongest ERD patterns. In addition, according to the comparison of ERSP maps and topographic distributions, MI-P produced weaker ERD patterns than any other paradigms, indicating that the illusion-based MI guidance systems amplified ERD well compared to pure MI.

The relative ERD results also show that the proposed MI guidance system enhanced MI to a greater degree than MI-P and at a similar level as RHI-P and ME-P. In the comparison of the ERD components in the contralateral region (Figure 5a), first, the finding that RHI-P amplified ERD significantly more than MI-P supports the results of our previous study [20]. Moreover, in this study, we found evidence that the proposed VHI-P also enhanced ERD to a significantly greater extent than MI-P, and would be similarly effective for MI enhancement as RHI-P. The peak amplitude and latency results showed that the ERD of VHI-P was more similar to that of ME-P than that of the other paradigms. From the viewpoint of peak amplitude, RHI-P also showed similar results to VHI-P and ME-P. Based on these results, we believe that body ownership of the virtual hand can be induced, and the guidance system using VHI improves the user’s MI ERD to a similar extent as the user’s ME ERD. Furthermore, as BCI input signals and signal features affect movement classification as well as accuracy [42,43,44,45], enhanced signal features (i.e., peak amplitude) might increase the accuracy of classification in MI-BCI. This illustrates that our proposed MI guidance system, VHI-P, as well as RHI-P can solve the issue of weak signals in MI paradigms which lead to the poor performance of MI-BCI. 

Unlike the results from the contralateral region, the peak ERD amplitude from the ipsilateral region was close to being statistically significant differences between all of the paradigms (*p* = 0.058). This resulted from differences in peak ERD amplitude between MI-P and ME-P. The results show that there is little difference in the ipsilateral region between all paradigms. Previous studies showed that ME induced stronger activation of ipsilateral region than MI as well as AO and MI, and AO did not produce significant changes in ipsilateral region in the motor area [46,47]; also, guided MI showed similar activation as MI, and only ME showed higher activation than MI. However, the latency from the ipsilateral region was similar to the latency from the contralateral region, and VHI-P and ME-P showed significantly lower latencies than RHI-P and MI-P in the ipsilateral region (Figure 5b). Since latency is one of the key elements related to fast detection in BCI, VHI-P also helps MI-BCI quickly detect a user’s MI and MI-BCI provides feedback to the user with low latency. As there was less variation in latency in VHI-P than in the other paradigms, we believe that VHI-P is a promising solution with low latency in all subjects.

In the questionnaire results (Figure 6), all subjects reported that the guidance system made it easier to concentrate on and imagine a given task. More users reported body ownership over the fake hand in VHI-P than in any other MI guidance system, and the number of subjects who showed larger (amplified) ERD in VHI-P compared to MI-P is greater than the number of subjects who showed larger (amplified) ERD in RHI-P, though the results of this questionnaire item were not always related to actual MI enhancement. However, as the questionnaire is subjective and included only one question related to body ownership, further studies on the relationships between body ownership, sense of agency, and actual MI enhancement are needed. Furthermore, although the time it takes to induce the illusion of body ownership varies from person to person [20,48], the present study produced the illusion within 3 min equally in all subjects and paradigms to enable a fair comparison. Fortunately, no subject reported that no illusion occurred, though the level to which body ownership is induced may vary by subject.

Our proposed system has relatively few physical constraints, which makes the system easy to extend to other body parts or target motions, because the rubber hand and brush were replaced with a virtual hand and motion tracking sensor. This is in contrast to previous MI guidance systems using RHI, which required physical objects, such as a motorized rubber hand and brushes, to induce the sense of body ownership and enhance the user’s MI [20]. There were several studies which reported VR-based AO using MI-BCI also could induce the body ownership without significant physical constraints [17,18,19]. However, they need the assumption that the user’s MI is well performed and enough to build MI-BCI classifier. Furthermore, their body ownership transfer could suffer from the mismatch between visual and proprioceptive information due to the discrepancy between virtual body parts and real body parts [25,49], while our proposed system has very little discrepancy between them. Moreover, in a previous study, the RHI-based MI guidance system showed significantly higher activation than pure MI and first-person perspective AO with VR. Although the present paper did not compare the proposed MI guidance system to AO-based MI guidance, the relationship between two MI guidance systems (i.e., AO-based versus VHI-P) can be indirectly compared to the relationship with RHI-P, through the previous study [20]. Therefore, since the proposed MI guidance system using VHI showed an MI enhancement effect that was comparable to that of RHI and had shorter latency than that of RHI, it could be the only system that has the dual advantages of ease of extension to other parts and better enhancement of MI. Moreover, the proposed MI guidance system has the potential for widespread use due to its versatility.

This study has some room for improvement. First, it is not clear whether the VR environment produced results that were similar to or better than the results of a physical system because there is a difference between the induction methods of the two illusion-based MI guidance systems, RHI-P and VHI-P. RHI-P induced the sense of body ownership through visuo-tactile information, while VHI-P induced it through visuomotor (visuo-proprioceptive) information [25,26,49]. Although there have been several previous studies that attempted to identify the type of information that would best induce the user’s sense of body ownership, a comparison of the MI enhancement effect between guidance systems using visuo-tactile information and visuomotor information has not yet been done. Next, that the proposed system requires the subject’s actual movement need to be improved for some BCI users who cannot move. Another limitation of our study is that we did not directly analyze the relationship between the type of MI guidance system and the classification results. As mentioned before, although amplification of signal features can lead to high classification performance [42,43,44,45], the direct relationship between the two should be proven in further studies. In addition, verification of the MI guidance system on external devices with BCI technologies, such as real-life appliances or assistant/rehabilitation robots, is required, and long-term studies that include the effects of training should also be done in future.

## 5. Conclusions

In this paper, we proposed an easily extendable MI guidance system and evaluated its capacity for MI enhancement. Whereas the state-of-art method of enhancing MI with a guidance system required a physical system to induce the user’s sense of body ownership, the proposed MI guidance system has few physical constraints thanks to its adoption of VR and motion tracking sensors. Despite having few physical constraints, the proposed MI guidance system with VHI showed significantly enhanced ERD compared to pure MI (*p* < 0.05) and similar ERD patterns as those produced by pure ME (*p* > 0.1) in terms of peak amplitude and latency. Hence, we believe that the proposed MI guidance system with VHI enhanced the MI signal and can be widely used for various applications with MI-BCI.

## Figures and Tables

**Table 1:** Statistical analysis for the contralateral and ipsilateral channel. Friedman test and Wilcoxon signed rank test between paradigms (asterisks indicate significant differences, * *p* < 0.05, ** *p* < 0.01).

		Friedman Test	Wilcoxon Signed Rank Test					
Channel	Parameter	*p*-Value						
			MI-RHI	MI-VHI	MI-ME	RHI-VHI	RHI-ME	VHI-ME
Contralateral channel	Peak ERD amplitude	0.008 **	0.016 *	0.016 *	0.003 **	0.722	0.790	0.534
	Latency	0.032 *	0.155	0.041 *	0.050 *	0.016 *	0.004 **	0.248
Ipsilateral channel	Peak ERD amplitude	0.058	0.091	0.594	0.033 *	0.722	0.182	0.110
	Latency	0.025 *	0.114	0.026 *	0.013 *	0.026 *	0.091	0.477
